# Self-perceived physical fitness as a moderating variable in the relationship between motor self-efficacy and physical self-concept in school-age physical education students

**DOI:** 10.3389/fpsyg.2024.1462333

**Published:** 2024-11-22

**Authors:** Carmen Galán-Arroyo, Noelia Mayordomo-Pinilla, Antonio Castillo-Paredes, Jorge Rojo-Ramos

**Affiliations:** ^1^BioẼrgon Research Group, Sport Sciences Faculty, University of Extremadura, Cáceres, Spain; ^2^Grupo AFySE, Investigación en Actividad Física y Salud Escolar, Escuela de Pedagogía en Educación Física, Facultad de Educación, Universidad de Las Américas, Santiago, Chile

**Keywords:** adolescents, physical education, quality of life, physical activity, mental health

## Abstract

**Introduction:**

The increasing prevalence of mental health disorders has elicited significant concern within society, particularly among adolescents who are disproportionately affected by this concerning trend. Consequently, institutions are seeking efficacious and cost-effective interventions to address this situation, while the scientific community has identified physical activity as a potential means to mitigate this epidemic. Motor self-efficacy and physical self-concept have been demonstrated to confer benefits to the mental health of young individuals, comparable to those of physical fitness. These aspects not only influence physical health but also the emotional and psychological well-being of individuals, contributing to a better overall quality of life. Objective: The aim of this study was to analyze the moderating role of self-perceived physical fitness in motor self-efficacy and physical self-concept, in high school physical education students.

**Methods:**

To this end, the Kolmogorov–Smirnov test was applied to identify the non-normality of the data and the Mann–Whitney U test to determine the differences by sex in the variables studied to subsequently perform a moderation analysis and a regression test for the physical self-concept prediction model.

**Results:**

The results showed that self-perceived physical fitness positively moderated the relationship motor self-efficacy and physical self-concept, enhancing the positive effect size of self-efficacy on physical self-concept.

**Conclusion:**

Thus, self-perceived physical fitness could be important in interventions aimed at improving physical and emotional well-being and health of adolescents.

## 1 Introduction

There is a wealth of evidence on the benefits of physical practice in different areas of health, encompassing physical, physiological, psychological, and even social health throughout all stages of life (Warburton and Bredin, 2017; Fitzgerald et al., 2022). At present, mental health has been gaining importance in society, allocating a greater amount of resources, and increasing research in this field, partly due to the existing pandemic related to mental disorders (Biddle et al., 2019). The World Health Organization (WHO) reports an upward trend among young people with this type of psychological condition: 14% of individuals between 10 and 19 years of age with this type of disorder, without taking into account many others that are often not identified due to the stigmatization of mental illness (World Health Organization, 2021). Adolescence is a difficult stage for young people because of the physical changes caused by sexual maturity that often provoke insecurities and comparisons with their peers, thus affecting their self-perception. Likewise, self-concept has been defined as a construct that combines the beliefs that an individual has about him/herself, influenced by previous experiences in a specific sociocultural context (Herrera et al., 2020; Montoya Londoño et al., 2019), with five subdivisions concerning academic labor, social, emotional, family, and physical spheres (García and Musitu, 2009). The study by Padial-Ruz et al. (2020) revealed that individuals who participated in physical activity exhibited higher levels of self-concept across all dimensions. This finding supports the beneficial impacts of physical activity on both physical and mental well-being, as well as social interactions, which are particularly pronounced during adolescence (Wickman et al., 2018). While some research on self-concept has demonstrated a stronger connection between physical activity engagement and enhanced physical self-concept, these studies failed to establish links with other self-concept dimensions (Christiansen et al., 2023). Physical activity has been demonstrated to have a positive effect on the self-concept of the adolescent population, regardless of gender, which occurs through the influence of physical activity on the physical dimension of physical self-concept (Fernández-Bustos et al., 2019). Physical self-concept encompasses motor performance and the way in which individuals perceive themselves physically; thus, different studies have found that a high self-concept is related to better self-esteem, motor perception and development, and participation in physical sports activities, which may be associated with a healthier lifestyle (Grao-Cruces et al., 2017; Kang et al., 2020; Navarro Patón et al., 2018; Videra-García and Reigal-Garrido, 2013).

Continuing with factors that could improve the mental health of adolescents, motor self-efficacy (MSE) refers to the feeling of competence in the face of the challenges that arise in people’s daily lives related to the perception of control and overcoming them with the aim of reducing the uncertainty caused (Hernández-Álvarez et al., 2011), specifically focusing on the motor field and the possibility of maintaining physical activity (PA) within daily habits. Related to these daily habits, Bandura (2004) identified this variable as a fundamental factor in the process of maintaining the decision-making process for participation in physical activity. In the case of adolescents, physical activity has also been recognized as an element that improves motor self-efficacy by increasing motor-related skills (Colella et al., 2020). This concept has been related to better experiences and greater enjoyment in physical education classes, increasing their performance in this subject, and improving participation rates in both school and extracurricular physical activities (Zhu and Chen, 2013). Moreover, a greater recognition and social support have been determined by some authors, suggesting a higher level of self-esteem and a lower rate of cyberbullying, accompanied by a lower number of disruptive behaviors (Kaefer and Chiviacowsky, 2021; Morales-Sánchez et al., 2021; Rojo-Ramos et al., 2024; Velázquez Buendía et al., 2015). In a study on the relationship between physical self-concept and PA, PA was found to influence physical self-concept by increasing self-esteem through a greater sense of motor skill (Schmidt et al., 2015).

In this sense, self-perceived physical fitness (PF), a parameter that, in addition to being a marker of health and a predictor of mortality (Mora et al., 2003), quantifies the physical abilities of individuals and allows them to overcome with greater ease the obstacles that may appear in daily life with vigor (Caspersen et al., 1985). Therefore, self-perceived physical fitness is a stable way of quantifying habitual physical activity levels (Blair et al., 2001). It can be divided into different dimensions, including cardiorespiratory capacity, muscular strength, agility, speed, and flexibility (Caspersen et al., 1985). In adolescents, associations have been identified with mechanisms that modify the state of health, both physically and mentally (Silverman and Deuster, 2014). Body composition is also related to physical condition (Caspersen et al., 1985), directly affecting self-perception, and therefore, determining self-esteem and self-perception (Streeter et al., 2012; Kołoło et al., 2018). Following this line of thought, good PF allows individuals to gain motor competence and increase motor self-efficacy and the enjoyment of PA, simultaneously increasing the practice of PA (Castelli and Valley, 2007), demonstrating that there is a correlation between both variables (Ericsson and Karlsson, 2011). On the other hand, self-perceived PF also has a close relationship with physical self-concept, with self-concept increasing with higher self-perceived PF, translating into greater self-esteem related to physical appearance and motor skills (Balsalobre et al., 2014).

After determining the relationships between these three variables and considering the problems regarding the mental health of young people, it is logical to look for tools that can be implemented to improve the status of mental health among adolescents. In this sense, the proposal of motor self-efficacy as a basis for improving physical self-concept is interesting, since through social recognition and a greater sense of ability, self-perception improves. To date, no study has been found that explored the role of self-perceived PF in this relationship. Considering the effects of self-perceived PF on both variables, the following question arises: does self-perceived PF influence the interaction between motor self-efficacy and physical self-concept? The aim of this study was to analyze the moderating role of self-perceived physical fitness between motor self-efficacy and physical self-concept.

## 2 Materials and methods

### 2.1 Participants

The total sample consisted of 1,231 students selected using the non-probabilistic convenience sampling method (Salkind, 1999). The sample was sex-balanced, with 49.1% (*N* = 604) males and 50.9% (*N* = 627) females. The mean age of the girls was 15.00 (SD = 1.90), while that of the boys was 14.00 (SD = 1.70). The mean age of the sample was 14.5 years (SD = 1.83). The inclusion criteria were as follows: (a) they had informed consent from their parents or legal guardians; (b) they were students of Physical Education in public or private-subsidized schools in Extremadura at the Secondary Education stage; (c) they are between 12 and 18 years.

This study was conducted in accordance with the ethical principles of the Declaration of Helsinki, and the protocol was approved by the Bioethics Committee of the University of Extremadura (Register Code 71/2022) (Table 1).

**Table 1:** Sample characterization (*N* = 1,231).

Variable	Categories	*N*	%
Sex	Boys	604	49.1
	Girls	627	50.9
**Variable**		**M**	**SD**
Age	Boys	14.0	1.70
	Girls	15.0	1.90

N, Number; %, Percentage; SD, Standard deviation; M, Mean.

### 2.2 Procedure

To recruit the necessary participants, the directory of the Department of Education and Employment de la Junta de Extremadura was consulted to obtain the contact details of the educational institutions between 12 and 18 years of age, in which their students participated in physical education. Once the contact data had been collected, information on the research was sent to the directors of the schools, explaining the procedure, the objective, and a copy of each evaluation instrument, in addition to the sociodemographic questionnaire and informed consent form. Subsequently, once the collaboration was established, this information was passed on to the physical education teacher to arrange an appointment in which a member of the research team would come to the school to apply the questionnaires in the presence of the teacher. Once informed parental consent was collected, the researcher proceeded to provide each student with a tablet with a link to the questionnaires, reading each item aloud to explain each element of these tools to solve any doubts the students might have and to ensure that all had understood the content of the questionnaires. The selection of electronic devices for data application, storage, and processing was driven by the desire to conserve material resources and reduce time expenditure. All data were stored anonymously between September and December 2022, taking approximately 10 min to complete the questionnaires.

### 2.3 Instruments

#### 2.3.1 Sociodemographic data

A questionnaire was prepared with two questions aimed at determining the characteristics of the sample: sex (male/female), age (years).

#### 2.3.2 Visual analogical scale of physical fitness perception for adolescents

In order to quantify self-perceived PF, this visual scale was applied to identify the dimensions of this characteristic: general physical condition, cardiorespiratory fitness, muscular strength, speed-agility and flexibility (i.e., “my global physical fitness is…”) (Mendoza-Muñoz et al., 2021). The instrument uses a Likert scale of to 1–10, with 1 = very poor and 10 = very excellent. The authors found that the psychometric properties of the instrument fit the model, with a Cronbach’s alpha of 0.860 with fit values obtained through Confirmatory Factor Analysis such as X^2^ = 0. 433; df = 24; *p* < 0.001; CFI = 0.999 RMSEA = 0.016; SRMR = 0.036. Subsequently, in this study, Cronbach’s alpha and McDonald’s Omega tests were replicated to obtain satisfactory values, according to Nunnally and Bernstein (1994).

#### 2.3.3 Motor self-efficacy scale

This instrument was applied in a validated Spanish version (Hernández-Álvarez et al., 2011). Its quantification system is based on a Likert scale with values from to 1–4, where 1 is “totally disagree” and 4 is “totally agree,” with a total of 10 items related to situations that can occur in sports practice (i.e., “during a sports game, I can manage to solve a problem even if someone opposes me”). This scale reported good self-efficacy and higher scores. The authors reported satisfactory psychometric characteristics, Kaiser Meyer-Olkin sample adequacy (KMO = 0.90) with a significant Bartlett’s test of sphericity (X^2^ = 5223.8; *p* = 0.000) and a Cronbach’s alpha of 0.89.

#### 2.3.4 Self-concept scale AF-5

Self-concept scale AF-5 composed of five dimensions divided into six items each, constituting a total of 30 items referring to different life contexts: academic work, social, emotional, family, and physical (García et al., 2011). In this study, only the last dimension related to PA was analyzed (i.e., “I am good at practicing sports”). The scores were obtained using a Likert scale from 1 to 5, where 1 was “never” and 5 was “always.” The psychometric properties of this scale in Spanish adolescents were acceptable, with the following data: KMO = 0.859, CFI = 0.99, GFI = 0.95, RMSEA = 0.029, and RMSR = 0.067, showing that the instrument fit the model (Zurita-Ortega et al., 2021).

### 2.4 Statistical analysis

First, the distribution of the data was examined to determine whether the assumption of normality was met to determine the type of statistical tests to be used. The Kolmogorov–Smirnov test was used for this purpose. The result of this test showed that this assumption was not met (*p* < 0.005). Therefore, nonparametric statistical tests were performed. The Mann–Whitney U test was used to analyze the differences in the scores obtained on the physical self-concept, motor self-efficacy, and self-perceived PF scales according to the sex of the participants. A significance value of *p* < 0.05. Subsequently, Hedges’ g was applied to determine the effect size of sex on the motor self-efficacy, AF-5, and FPVASA scales. Values less than 0.20 indicate no effect, values between 0.21 and 0.49 indicate a small effect, values between 0.50 and 0.80 indicate a moderate effect, and finally, values greater than 0.80 indicate a strong effect (Cohen, 2013). In addition, a moderation analysis was performed to explore whether self-perceived PF modified the relationship between motor self-efficacy and physical self-concept.

To conduct an in-depth analysis of self-efficacy, a stepwise regression test was performed by entering the significant variables into the prediction model. Finally, Cronbach’s Alpha and McDonald’s omega coefficients were used to evaluate the reliability of the psychometric scales from their internal consistency and to interpret the reported values; those established by Nunnally and Bernstein were chosen. The maximum likelihood model (Omega ML) was used to calculate the McDonald’s Omega coefficient.

The data are presented as the number and percentage for the sociodemographic variables and as the mean (M) and standard deviation (SD) for scores obtained in each of the scales. The software used for data analysis was JAMOVI, version 2.4.8.0.

## 3 Results

Table 2 shows the results of the analysis by sex of the variables studied in this study, revealing that there were statistically significant differences in all of them, with boys expressing a higher level of self-perception of PF, physical self-concept, and motor self-efficacy. In terms of effect size, sex showed a moderate effect in the case of physical self-concept (*g* > 0.50), the same as motor self-efficacy. In the case of self-perceived PF, the effect size was small, but close to a moderate magnitude.

**Table 2:** Descriptives, differences, effect size, and relationship between AF-5, VAS PFA, and motor self-efficacy.

Variable	Total	Sex			
		Boy	Girl		
	M (SD)	M (SD)	M (SD)	*p*	g
AF-5 (physical dimension)	3.51 (0.78)	3.75 (0.74)	3.28 (0.75)	<0.001	0.626
VAS PF A	6.66 (1.62)	7.01 (1.44)	6.32 (1.71)	<0.001	0.433
Motor self-efficacy	3.11 (0.62)	3.28 (0.56)	2.95 (0.64)	<0.011	0.545

*p* is significant < 0.05*. M, Mean; SD, Standard deviation; g, Effect size.

### 3.1 VAS-PFA moderation analysis

A moderation analysis (Table 3) was performed to determine the role of self-perceived PF in the relationship between motor self-efficacy and physical self-concept. The results indicated a significant positive effect of motor self-efficacy on AF-5 (*β* = 0.035, SE = 0.002, *p* < 0.001). Similarly, self-perceived PF had a significantly greater effect on physical self-concept (Î^2^ = 0.240, SE = 0.009, *p* < 0.001). Significantly, the interaction between motor self-efficacy and self-perceived PF resulted in a positive moderating effect (*β* = 0.003, SE = 0.001, *p* = 0.018), albeit with reduced magnitude.

**Table 3:** VAS PF A moderation analysis.

	β	SE	Boot 95% CI		Z	*p*
			LL	UL		
Motor self-efficacy	0.035	0.002	0.030	0.041	13.21	<0.001
VAS	0.240	0.009	0.220	0.259	24.08	<0.001
Motor self-efficacy* VAS	0.003	0.001	5.50e-4	0.005	2.37	0.018

The prediction model for physical self-concept, presented in Table 4, revealed that a model composed of motor self-efficacy, subjective rating of self-perceived PF, and sex could explain 49% of the variance in physical self-concept (*R*^2^ = 0.49). This robust model identified motor self-efficacy (*β* = 0.310, *p* < 0.001) and VAS PFA (*β* = 0.229, *p* < 0.001) as significant predictors of physical self-concept, with both showing a positive relationship. Sex emerged as a significant predictor with a negative effect (*β* = −0.208, *p* < 0.011).

**Table 4:** Physical self-concept prediction model.

	Model 1 (*R*^2^) = 0.49			
Variable	β	SE	*t*	*p*
Motor self-efficacy	0.310	0.032	9.585	<0.001
VAS PFA	0.229	0.012	18.515	<0.001
Sex	−0.208	0.033	−6.244	<0.011
Constant	1.231	0.161	7.637	<0.001

The reliability of the instrument was calculated based on the internal consistency of the three instruments using Cronbach’s alpha and McDonald’s omega statistics (*ω* = 0.747). These values can be considered satisfactory, according to Nunnally and Bernstein (1994), shown in Table 5.

**Table 5:** Reliability analysis.

Scale	Cronbach’s Alpha	McDonald’s Omega
AF-5 (physical self-concept)	0.763	0.766
PF VAS A	0.777	0.803
Motor self-efficacy	0.907	0.907

## 4 Discussion

This study aimed to explore the role of self-perceived PF in the relationship between motor self-efficacy and physical self-concept in secondary school physical education students, exploring the effect of self-perceived PF on the interaction of both variables. Additionally, a prediction model was proposed that included significant variables that explain the variability of physical self-concept.

In the study of sex covariates in physical self-concept, motor self-efficacy, and self-perceived PF, statistically significant differences were obtained that also revealed a moderate effect size in the case of self-concept and self-efficacy, revealing importance of sex in this area. Its importance lies in the need to address sex as a differentiating characteristic between self-concept and self-efficacy when planning intervention programs or even in the planning of physical education in order to ensure a more equitable development of the perception of both variables. These results are consistent with those previously published in the scientific literature, revealing that boys perceive greater motor self-efficacy (Spence et al., 2010), probably due to greater PA practice, in the same way as self-perceived PF (Biadgilign et al., 2022). Contrary to these results, others found no significant sex-related differences, possibly due to the age of the sample being younger than the participants in this study, suggesting that social expectations often focus on the male sex as related to motor efficacy, building this confidence as they become adolescents (Grant-Beuttler et al., 2019; Muñoz and García, 2013). Mixed results appeared in the case of physical self-concept. Some studies claim differences in the perception of self-concept in favor of the male sex (Madrona et al., 2020; Rodríguez Villalobos et al., 2023), while others find no significant differences despite the fact that girls, in this case, perceive the subdimensions related to physical appearance to a greater extent (Planinšec and Fošnarič, 2005; Sáez et al., 2020), This suggests that self-concept varies depending on the environment in which an individual develops.

The results of the analysis of the interaction between the variables highlighted the significance of motor self-efficacy and self-perceived PF with physical self-concept, suggesting that higher levels of motor self-efficacy are associated with a more positive perception of self-concept, in addition to highlighting the importance of self-perceived PF as a predictor since it reveals a higher magnitude than motor self-efficacy in this association. In general terms, when a skill is mastered, there is a tendency to develop a greater perception of physical self-concept by increasing motor self-efficacy, extolling its individual characteristics and increasing self-confidence, facilitating recovery after failures in this context and not associating them with a lack of skill, in addition to the influence that this mastery has on self-esteem, mediated through physical acceptance (Padial-Ruz et al., 2020; Morales-Sánchez et al., 2021; Arribas-Galarraga et al., 2020). In this regard, the exercise and self-esteem model (EXSEM) reveals two levels of physical perception: a more general one related to physical self-worth and a specific one referring to sports competence, self-perceived PF, physical appearance, and strength (Sonstroem and Morgan, 1989). In line with self-perceived PF, a review with meta-analysis presented that those who experience an improvement in their self-perceived PF also obtain an improvement in their self-esteem through changes in physical self-concept (Babic et al., 2014); and that a high PF provides individuals with a greater range of improvement in their skills, feeling more competent in this sense (Balsalobre et al., 2014; de Benitez-Sillero et al., 2023), similar to what was obtained in this work. On the other hand, the interaction of self-perceived PF with the relationship between motor self-efficacy and physical self-concept turned out to be positive, suggesting that self-perceived PF influences the degree to which motor self-efficacy affects physical self-concept. This finding reveals that greater self-perceived PF would enhance the interaction of motor self-efficacy on physical self-concept, which follows the logic of models proposed by other authors, where greater skill in a PA would increase motor self-efficacy, which in turn raises self-belief about its worth and characteristics. This increase in self-efficacy may be due to good self-perceived PF, as multiple studies have revealed an association between motor self-efficacy and PA practice (Carraro et al., 2010; García et al., 2014). In addition, good self-perceived PF achieved through PA practice improves the perception of physical appearance, improving their acceptance at this life stage when their bodies change rapidly (Fernández-Bustos et al., 2019).

In a complementary manner, significant variables were introduced into the prediction model, identifying motor self-efficacy, self-perceived PF, and sex as predictors of physical self-concept. In this regard, the scientific literature reveals disparate results related to sex, with no significant differences found in this regard (Planinšec and Fošnarič, 2005; Sáez et al., 2020). In contrast, a meta-analysis carried out by Babic identified sex as a moderator in the relationship between physical activity and physical self-concept, especially in boys (Babic et al., 2014).

### 4.1 Practical applications

Self-perceived PF is an important predictor and moderating factor of motor self-efficacy in its relationship with physical self-concept, highlighting the importance of including self-perceived physical fitness in interventions aimed at improving physical self-perception through motor self-efficacy, considering the sex of the students. The differences found in this study reveal a different tendency in the perceptions of the three variables when studying sex.

### 4.2 Limitations and future lines

Similar to other studies, this study has certain limitations. It was designed to obtain the sample under convenience sampling; therefore, it was not possible to determine causality. In addition, the physical condition was obtained using a self-reported instrument, which, despite having good reliability, would have a lower risk of bias if they were objective measures. In addition, the sample comprised 12–18 years, a vital period where changes occur at a rapid rate and do not occur in the same way in both sexes; therefore, these results may be biased by the wide age range. In future research, it would be interesting to determine which dimensions (s) of self-perceived PF have a greater influence on the interaction of motor self-efficacy and physical self-perception. Additionally, it would be interesting to explore differences in motor self-efficacy and physical self-perception by studying the location and type of school, so that the environmental context can be addressed.

## 5 Conclusion

This study provides valuable insights into the complex relationships between motor self-efficacy, self-perceived PF, and physical self-concept among secondary school physical education students. These findings highlight the significant role of self-perceived PF in moderating the relationship between motor self-efficacy and physical self-concept, demonstrating that higher levels of self-perceived PF enhance the positive impact of motor self-efficacy on physical self-concept. Additionally, the prediction model identified motor self-efficacy, self-perceived PF, and sex as key predictors of physical self-concept, emphasizing the importance of considering sex as a covariate. The study’s results also underline the need to address sex differences in self-concept and self-efficacy when designing intervention programs in physical education. Statistically significant sex-related differences were observed, particularly in motor self-efficacy and physical self-concept, suggesting that boys may develop higher motor self-efficacy because of greater PA participation. These findings reinforce the idea that physical self-concept varies according to environment and sex-related factors, as supported by previous research. Overall, this study contributes to the body of knowledge by confirming the interplay between motor self-efficacy, self-perceived PF, and physical self-concept, while also stressing the importance of sex as a differentiating factor. The results align with the existing literature and highlight the potential for targeted physical education interventions to foster more equitable self-concept development across sexes, ultimately promoting positive self-perceptions and well-being in adolescents.

## Data Availability

The raw data supporting the conclusions of this article will be made available by the authors, without undue reservation.
